# Identification of cross-stage, cross-species malaria CD8^+^ T cell antigens

**DOI:** 10.21203/rs.3.rs-6682089/v1

**Published:** 2025-05-30

**Authors:** Camila R. R. Barbosa, Luna B. de Lacerda, Paulo Bettencourt, David Morrow, Dhelio B. Pereira, Maya Aleshnick, Julie Mitchell, Zezhou Zhao, Cristopher Gomes, Guilherme C. Maia, Gregório G. Almeida, Camila M. Costa, Annalisa Nicastri, Marie Rose Schrimpf, Roxanne Beebe, John B. Schell, Payton Kirtley, Lis Antonelli, Gaurav Das Gaiha, Scott Hansen, Klaus Frueh, Judy Lieberman, Ricardo T. Gazzinelli, Nicola Ternette, Brandon Wilder, Adrian V.S. Hill, Caroline Junqueira

**Affiliations:** 1 Instituto René Rachou, Fundação Oswaldo Cruz, Belo Horizonte, MG 30190-002, Brazil; 2 Centro de Tecnologia em Vacinas, Universidade Federal de Minas Gerais, Belo Horizonte, MG 31310-260, Brazil; 3 Program in Cellular and Molecular Medicine, Boston Children’s Hospital, Boston, MA 02115, USA; 4 Department of Pediatrics, Harvard Medical School, Boston, MA 02115, USA; 5 The Jenner Institute, University of Oxford, Oxford OX3 7DQ, UK; 6 Vaccine and Gene Therapy Institute, Oregon Health & Science University, Beaverton, OR 97006, USA; 7 Centro de Pesquisas em Medicina Tropical de Rondônia, Porto Velho, RO 78909-350, Brazil; 8 Ragon Institute of Mass General, MIT, and Harvard, Cambridge, MA 02139, USA; 9 Departmaneto de Bioquímica e Imunologia, Instituto de Ciências Biológicas, Universidade Federal de Minas Gerais, Belo Horizonte, MG 31270-901, Brazil; 10 Institute for Research in Biomedicine, Università della Svizzera Italiana, Bellinzona, TI 6500 Switzerland; 11 Division of Infectious Disease and Immunology, University of Massachusetts Medical School, Worcester, MA, 01605, USA; 12 School of Life Sciences, University of Dundee, Dundee, DD5 1EH, Scotland, UK

## Abstract

Malaria is one of the most prevalent parasitic diseases in the world. In 2023, 263 million malaria cases were estimated worldwide^1^. Two species of *Plasmodium, P. falciparum* and *P. vivax*, cause most human malaria. Despite the licensing of two partially protective vaccines for *P. falciparum*, there is no vaccine capable of providing long-term control or elimination^2,3^. A major limitation for vaccine development is the lack of validated T cell epitopes for either species that could be targeted by vaccines. *P. vivax* is the most widespread human malaria parasite and is the major species causing malaria in the Americas and Asia while *P. falciparum* is more prevalent in Africa^1^. *P. vivax* exclusively infects reticulocytes in peripheral blood, which, unlike the mature erythrocytes infected by *P. falciparum*, still retain RNA and therefore retain host protein translation capabilities^4^. We previously reported that *P. vivax*-infected reticulocytes express the major human leukocyte antigen class I (HLA-I), which allows parasite sensing by CD8^+^ T cells and consequent killing of parasite-infected host cells and intracellular parasites^5^. Here we report by immunopeptidomic analysis the first unbiased identification of *Plasmodium spp*. antigens presented via HLA-I on infected reticulocytes. We identified 453 unique peptides that mapped to 166 different proteins. Most of these antigens were housekeeping proteins that are constitutively expressed at multiple stages of the parasite life cycle. Common peptides were presented in different individuals by the same or distinct HLA-ABC alleles as well as by non-classical HLA-E. Many peptide sequences were highly conserved in *P. falciparum* and *P. vivax*. The immunogenicity of the newly identified epitopes was validated in both *P. vivax*- and *P. falciparum*-infected patient samples. Furthermore, several of these antigens were immunogenic in the blood and liver of non-human primates following *Plasmodium* infection and attenuated parasite immunization. Two antigens were also the target of protective CD8^+^ T cell-mediated immunity in rodents. Thus, these antigens have potential for use in a cross-stage and cross-species malaria vaccine.

One of the greatest hopes for controlling the persistent malaria burden is to develop a vaccine capable of blocking transmission and durable infection. Most vaccine efforts have focused on eliciting antibodies (Abs) to *Plasmodium* proteins that block parasite invasion of hepatocytes or red blood cells (RBCs)^6,7^. Immunization with live attenuated parasites that elicits both neutralizing antibodies that block infection and an anti-malarial T cell response that can kill the parasite during its intracellular stages provides the highest levels of protection against malaria^7,8^. However, little is known about which of the thousands of potential parasite antigens can be recognized by T lymphocytes, making inducing T cells a major challenge.

Most malaria research efforts have focused on *P. falciparum* (*Pf)*, making *P. vivax* (*Pv)* malaria a truly neglected tropical disease, despite its health importance. Currently, two *Pf*-restricted malaria vaccines are recommended by the World Health Organization, RTS, S/AS01 and R21/Matrix-M^9^. Both are adjuvanted virus-like particle vaccines based on the *Pf* circumsporozoite protein (CSP), which function by eliciting neutralizing antibodies against *Pf*CSP^2,3^. These vaccines exclusively target the *Pf* pre-erythrocytic stage and prevent clinical malaria in children with varying levels of efficacy depending on geographic location and *Pf* strain, reaching ~30% of clinical protection for RTS,S^2^ and >70% for R21^3^. While long-term studies are ongoing, both vaccines demonstrate waning efficacy that requires booster doses. Vaccines based on attenuated live sporozoites are the only strategies that have demonstrated high levels of long-lasting sterilizing immunity. In addition to neutralizing antibodies, these vaccines induce robust CD8^+^ T cell and γδ T cell responses^7,8^. *Pv* vaccine progress has been more limited; efforts have mostly focused on employing *Pf*-orthologous antigens to target the pre-erythrocytic proteins *Pv*CSP and *Pv*TRAP, or elicit antibodies to malaria proteins that mediate *Pv* binding to reticulocytes (Retics), such as *P*v Duffy Binding Protein (*Pv*DBP) and *Pv* Merozoite Surface Protein 1 (*Pv*MSP1)^10^. An ideal vaccine would protect against both the liver and blood stages of infection for multiple years and target antigens with cross-species reactivity.

Previously, T lymphocytes were thought to only recognize the *Plasmodium* intracellular liver stage, because mature RBCs do not express the HLA molecules that present antigenic peptides to T lymphocytes. While *Pf* infects mature RBCs, *Pv* infects immature RBCs, reticulocytes^4^. We previously showed that CD8^+^ T lymphocytes recognize *Pv*-infected retics (iRetics), lyse the host cell and directly kill the parasite^5^—thus preventing it from infecting new cells and spreading the infection. Here we leveraged this discovery to identify *Pv* peptides presented by HLA-I on iRetics by using immunopeptidomics and mass-spectrometry to uncover an array of putative T cell malaria epitopes. Importantly, most of the 166 identified antigenic proteins are expressed in all parasite stages and are highly conserved across different *Plasmodium* species. Indeed, cross-species recognition was confirmed for 30 of 50 peptides tested. These epitopes were immunogenic in *Pv*- and *Pf*-infected patients, *P. cynomolgi* (*Pcy*)- and *P. knowlesi (Pk)*-infected non-human primates (NHP) and *P. yoelii (Py)*-infected mice. Based on these exciting data, we hypothesize that these broadly recognized *Plasmodium* T cell antigens could be used to design a universal cross-species malaria vaccine that induces T cell-mediated immunity against liver and blood stage parasites.

## Identification and characterization of HLA-I-bound *Pv* peptides

**Figure 1 |:**
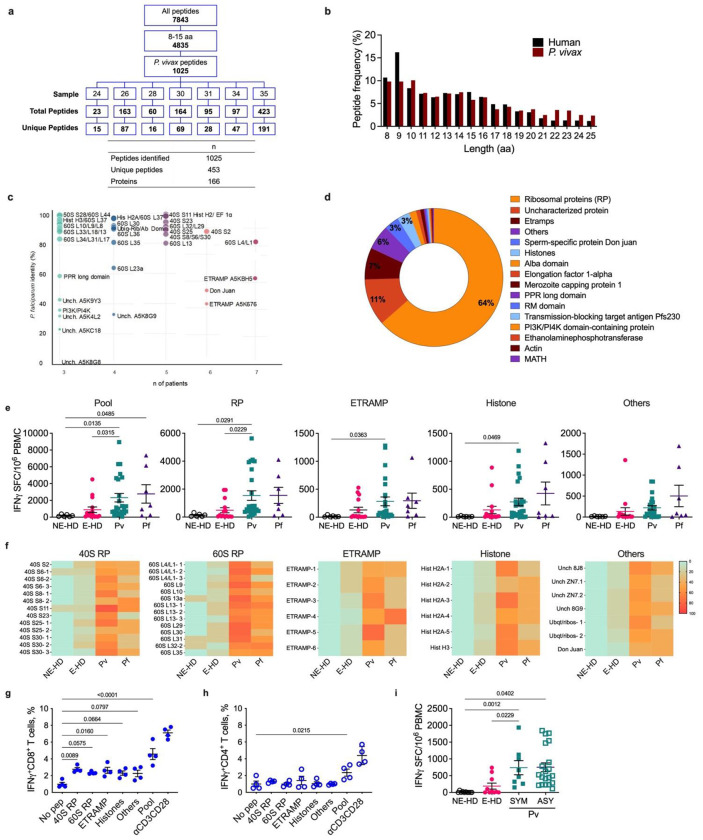
Peptide identification and immunogenicity validation. **a,** Summary of peptides identified by LC-MS/MS using databases containing the human (Uniprot- UP000005640 - Version 09-2019) and *P. vivax* (*Pv*) Sal-1 (Uniprot- UP000008333- v 08-2019) proteomes. Unique peptides across the samples were selected according to size (between 8 and 15 amino acids) and presence in the predicted *Pv* proteome but not human proteome. **b,** Frequencies (in percent of total) of peptide length within unique human and *Pv* peptides spanning from 8 to 25 amino acids. **c,** Antigens found in at least 3 patients were plotted by their percent identity to their respective *P. falciparum* (*Pf*) orthologs versus the number of patients in which they were found (indicated by colour intensity where light blue is the minimum and dark pink the maximum) and identity to *Pf* by dot diameter (the larger the diameter, the greater the shared identity). **d,** Frequencies of the protein families to which the identified *Pv* proteins belong. **e-f,** ELISpot results for selected peptides tested using PBMC isolated from patients infected with *Pv* (n=24), *Pf* (n=7), or healthy donors from malaria endemic (E-HD; n=15) and non-endemic (NE-HD; n=6) regions. **e,** PBMC were stimulated with a pool of each of 50 peptides **or** pools of peptides derived from ribosomal proteins (RP), early transcribed membrane proteins (ETRAMP), histone and other proteins. IFNγ production was measured by ELISpot and the results are expressed as spot-forming cells (SFC) per 1x10^6^ PBMC. **f,** The percentages of positive individuals for each peptide tested by each infected or healthy group are depicted by colour intensity. Green, orange and red represent low, middle, or high levels of responders in the ELISpot assay, respectively. **g-h,** Peptide pools prepared according to the antigen family group were used to stimulate PBMC from *P. vivax* symptomatic patients (n=4). Frequencies of IFNγ producing CD8^+^ T cells (**g**) or CD4^+^ T cells (**h**) were determined by flow cytometric intracellular cytokine staining. αCD3 and αCD28 monoclonal antibodies were used as positive control and a no-peptide condition as negative control. **i,** ELISpot results obtained with PBMC from E-HD (n=10), NE-HD (n=10), *Pv* symptomatic (SYM; n=8) and *Pv* asymptomatic (ASY; n=20) patients upon stimulation with a pool of all 50 peptides. **e, g-i,** Statistical analyses were performed by one-way ANOVA. Comparison across multiple groups was performed by Kruskal Wallis test. Actual P values are shown.

To identify malaria peptides that bind to HLA-I molecules in *Pv*-infected retics, we obtained peripheral blood samples from 7 acute malaria patients (SI. 1) from Porto Velho, Rondônia, a malaria endemic region in Brazil. Patient samples were anonymized as 24, 26, 28, 30, 31, 34, and 35. Highly purified iRetics contained 4.3x10^7^ to 7x10^8^ cells and were more than 99% infected. After applying an optimized protocol pipeline of HLA-peptide complex purification followed by mass spectrometric analysis of each sample^11^, a total of 7,843 peptides were identified, of which 4,835 had an HLA-I peptide size distribution, ranging from 8-15 amino acids. 1,025 peptides matched to the *Pv* proteome (Proteome ID: UP000305196). The number of peptides per sample ranged from 23 to 423 peptides (Fig. 1a). Combining all samples, we identified 453 unique *Pv* peptides from 166 proteins (Fig. 1a, SI. 2). The length distribution of the 8-25mer peptides indicated a typical HLA class I- restricted profile. 9-mer peptides were the most abundant human peptides. *Pv* peptides showed a high frequency of 8-10-mers, but there were many longer peptides especially 10-16-mers (Fig. 1b).

Sequence comparison between *Pv* and *Pf* proteomes showed that 34 of the *Pv* peptide-source proteins share more than 80% identity with *Pf* proteins (Fig. 1c - y axis, Extended data table 1). Importantly, identical peptide sequences were found in samples from different patients. Figure 1c – x axis shows proteins that had the same peptide in 3-7 samples from the 7 patients. Peptides from ribosomal protein (RP) 60S L4/L1 and ETRAMP (A5KBH5) were found in all 7 samples. These proteins share 83% and 63% amino acid sequence identity with *Pf* proteins, respectively. ETRAMP (A5K676), RP 40S S2 and Don Juan protein were represented by peptides in 6 of 7 samples and exhibited 43%, 97% and 53% *Pf* homology, respectively. Peptides from 11 proteins were found in 5 of 7 samples, the majority of which were from the 40S RP family with 80-100% homology to *Pf*. Peptides from 9 proteins were identified in 4 samples (*Pf* homology: 33-97%) and peptides from 20 proteins were found in 3 of 7 samples (*Pf* homology: 0-97%). Analysis of the mass spectrometry data showed that of 166 *Pv* source proteins found in all samples, the vast majority were RP (64%), followed by uncharacterized proteins (11%), Early transcribed membrane protein – ETRAMP (7%), other proteins (6%), Sperm-specific protein Don Juan (3%) and histones (3%) (Fig. 1d).

As most of the identified peptides were from housekeeping proteins, we investigated whether these peptides are also expressed during the sporozoite and liver stages of the parasite life cycle using published transcriptomic data for the *Pv* blood (*Pv*Sal1 strain)^12–14^, liver (*Pv*P01 strain)^15^, and sporozoite (*Pv*Sal1 strain)^16^ stages. All 166 proteins are indeed expressed during the blood stage, 153 proteins are expressed during *Pv* liver stage, 146 proteins are expressed in the liver dormant form –hypnozoites, and 125 proteins are expressed by sporozoites (SI. 3). Likewise, *Pf* transcript orthologs were searched in the plasmoDB databank^17^, Malaria Cell Atlas^17^ and published data for *Pf*-infected hepatocytes^18^. 143 transcript orthologs are expressed during *Pf*3D7 blood stage, 143 in liver stage and 135 in sporozoites (SI. 3). In addition, relative gene expression of a subset of these transcripts was confirmed by RT-qPCR using liver samples from humanized mice infected with *Pf* sporozoites (Extended data fig. 1). Expression of CSP and TRAP were measured as positive controls. All genes that were tested were found to be expressed during the *Pf* liver stage. 40S RP S25, 40S RP S30 and 60S RP L29 were highly expressed similarly to the positive controls, while 60S RP L13 and Histone H2A had higher expression than CSP and TRAP.

## CD8^+^ T cells from *Pv*- and *Pf*-infected patients respond to *Pv*-derived HLA-associated peptides

To determine whether the identified peptides were epitopes recognized by patient T cells, 50 peptides were selected for further study based on the following criteria: i) originating from source proteins found in at least 3 patients; ii) the same peptide sequence was found in multiple patients; iii) peptides with the highest Peaks score. All 50 peptides (SI. 4) were synthesized and tested by spectral match validation to compare to the original biological *Pv* peptides (Extended data fig. 2), prior to immunological assessment. Forty-four of the 50 selected peptides were confirmed for spectral match.

Individual peptides, classified as ribosomal, ETRAMP, histone and other peptides, were tested in anti-IFNγ ELISpot assays using peripheral blood mononuclear cells (PBMC) from *Pv*- and *Pf*-infected patients, or healthy donors (HD) from endemic (E-HD) and non-endemic (NE-HD) regions (Fig 1e, f, Extended data fig. 3). The mean number of IFNγ-producing spot-forming cells (SFC) from samples stimulated with the pool of 50 peptides was significantly higher in *Pv*-infected patients as compared to E-HD or to NE-HD, and in *Pf*-infected patients compared to NE-HD. (Fig 1e). There was no difference in the IFNγ-producing SFCs in samples from *Pv*- and *Pf*-infected patients. The same pattern was observed in the ELISpot results of ribosomal, in which samples from *Pv*- and *Pf*-infected patients showed similar responses, *Pv*-infected patients had significantly higher responses when compared to E-HD or to NE-HD, and *Pf*-infected patients were different to NE-HD (Fig 1e). Samples from *Pv*-infected patients responded significantly more to ETRAMP, and histone peptides than NE-HD. Other peptides had no significant difference across the tested groups. *Pf*-infected patients displayed an elevated, but not statistic different, number of SFC when stimulated with ETRAMP, histones and other peptides. This statistical analysis and also the comparations from *Pf* to E-HD in pool and ribosomal groups were possibly affected by the small number (n=7) of *Pf*-infected patients. Extended data fig. 3 shows the SFC for each individual peptide in *Pv, Pf*, E-HD and NE-HD.

The ELISpot assay for each protein group showed a high frequency of responders to both 60S ribosomal and 40S ribosomal in *Pv- and Pf*-infected patients (Fig 1f). The proportion of *Plasmodium*-infected patients who responded to ETRAMP and histone peptides was more moderate. No NE-HD samples responded to the tested peptides, while some E-HD with a previous history of *Pv* infection produced IFNγ in response to some of the peptides (Fig 1e, f). In general, the

Next, the ELISpot assays of IFNγ production by *Pv*-infected patient PBMCs to the pooled malaria peptides was also confirmed by flow cytometry. CD8^+^ T cells from *Pv*-infected patients produced significantly more IFNγ after stimulation with the pooled peptides, 40S ribosomal (40S), 60S ribosomal (60S), ETRAMP, histone, or other peptides than cells incubated without peptide (no pep) (Fig. 1g). In contrast, CD4^+^ T cells responded significantly only to the pool of all peptides (Fig. 1h). The reduced response of CD4^+^ T cells was expected since the peptides were isolated from HLA-I that is recognized by CD8^+^, but not CD4^+^, T cells.

In our actively screened study population, about 1/3 of the *Plasmodium*-infected patients (mainly *Pv*) present with subpatent parasitemia and are asymptomatic, possibly due to naturally acquired immunity^19–21^. To further characterize the relationship between immunoreactivity to the tested peptides and the absence of symptomatic malaria, we investigated the ability of PBMC from asymptomatic individuals infected with *Pv* to respond to the pool of 50 synthetic peptides (Fig. 1i). While NE-HD or E-HD donors had no or low responses to the pool, both symptomatic (SYM) and asymptomatic (ASY) showed a similar significant increase in IFNγ-SFC after stimulation with the peptide pools.

## *Pv*-derived peptides are presented by classical and non-classical HLA-I

We were surprised that many of the peptides were recognized by many of the subjects. To determine if the malaria peptides and proteins were presented by different HLA alleles, we HLA typed each of the 7 *Pv*-infected donors (Extended data fig. 4a). The NetMHCpan 4.0^22^ program was used to predict how many of the 50 chosen peptides were likely to bind to the classical HLA-A, B and C molecules of each donor (Extended data fig. 4b). A threshold of 10% was used to select binding peptides. Six of the 7 patient donor classical HLA molecules were predicted to to bind to multiple peptides (from 5-40 each), but one patient sample (#24) did not have any predicted binding peptides (Extended data fig. 4c). The predicted peptide lengths ranged from 8-15-mers, but most were 8-10-mers.

**Figure 2 |:**
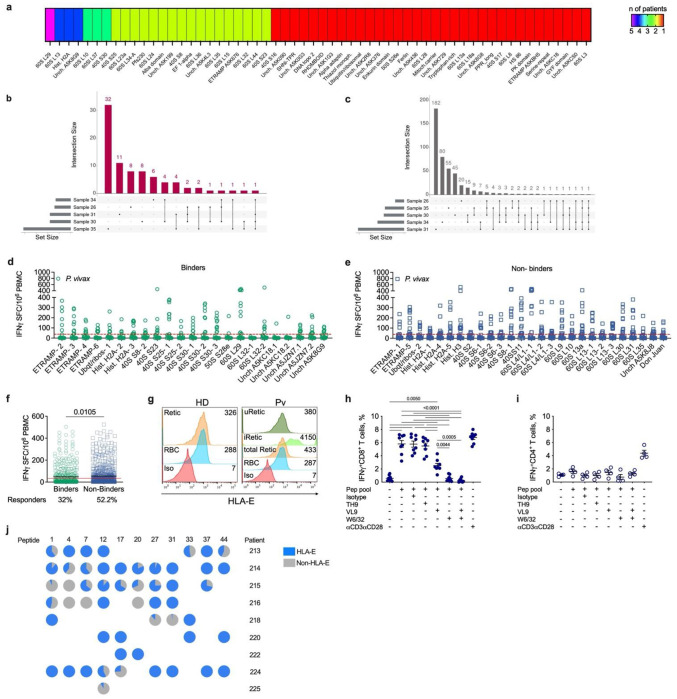
Classical and non-classical HLA peptide binders. **a,** Proteins containing peptides predicted to bind with high affinity to HLA-A, B or C alleles according to NetMHC. The color intensity represents the number of patients for whom peptides were predicted to bind to HLA-A,B or C (violet: maximum; red: minimum). **b-c,** UpSet plots visualizing the intersections of peptides found in different samples. Each column corresponds to a specific section in a traditional Venn diagram. Each row corresponds to one sample. The number of total peptides in the respective sample is indicated by horizontal bars. Connected, dark circles in the matrix indicate samples that share the same peptides (**b,** *P. vivax* (*Pv*); **c,** human). **d-f,** Peptides tested by ELISpot assay in *Pv*-infected patients were segregated into binders (**d**) or non-binders (**e**) based on NetMHC analysis. IFNγ production was measured by spot counting and the results are expressed as spot-forming cells (SFC) per 1x10^6^ PBMC. Responses to peptides were considered positive when ≥30 spots were recorded, as indicated by the red threshold line. **f,** Comparative analysis of IFNγ ELISpot results between peptides that bind (binders) and do not bind (non-binders) to HLA-A, B or C. **g,** Representative histogram of HLA-E staining on uninfected reticulocytes (uRetic) and erythrocytes (uRBC) from a healthy donor (HD) or a *Pv*-infected patient, and iRetic from the same patient. Median Fluorescence Intensity (MFI) is shown in the upper right of each histogram. **h-j,** PBMC from *P. vivax*-infected patients (n=8) were incubated with different combination of blocking reagents for HLA-E (VL9 peptide) and for pan HLA class I mAb (W6/32), or their negative controls (TH9 peptide or isotype mAb, respectively) prior to stimulation with a pool of all 50 peptides (**h, i**) or single peptides (**j**). Conditions without peptide were included as a negative control. aCD3 and aCD28 mAbs were used as a positive control. IFN γ production by CD8^+^ T cells (**h, j**) or CD4^+^ T cells (**i**) was determined by intracellular cytokine staining. **j,** the pie charts show the proportion of HLA-E-dependent (i.e. VL9 blocked) and non-HLA-E-dependent responses across different patients for individual peptides. Statistical analyses were performed by Mann Whitney test (**f**) and by one-way ANOVA, with Kruskal Wallis test (**h-i)**. Actual P values are shown.

The donors expressed a variety of distinct HLA haplotypes. Interestingly, many of the same or nested peptides were predicted to bind to different HLA alleles (SI. 5). For example, peptides from the 60S RP L29 had predicted high affinity to alleles from 5 different donors. 60S RP L13, histone H2A and uncharacterized protein (A5K8G9) peptides were predicted to bind to 4 different patients. Peptides from 60S RP L10, 60S RP L37 and 40S RP S30 were predicted to bind to 3 donors. Seventeen antigens had predicted high binding affinity for HLA-ABC alleles from 2 different donors (Fig. 2a, SI. 5).

Intersection analysis was used to visualize which patients eluted the same peptide that was predicted to bind HLA-ABC. Two *Pv*-derived peptide sequences were isolated from four different samples, while two peptides were eluted from three distinct samples. Most of the peptides shared among the 7 samples were isolated from two different donors (Fig. 2b). In addition to *Plasmodium* peptides, some of the host-derived HLA-ABC-bound peptides were also isolated from multiple *Pv*-infected donors. Two human peptide sequences were identified in 5 of the 7 samples; 4 peptide sequences were found in 4 of 7 samples, and 6 sequences were found in 3 of 7 samples. Most of the peptides were identified in two distinct samples (Fig 2c).

The identification of shared peptide sequences in multiple donors could be due to contamination and co-precipitation of proteins or their degradation products during the HLA immunoprecipitation or to the presentation of identical peptide ligands by different or shared HLA alleles. To stratify our ELIspot data accordingly, we reanalysed the T cell responses and designated the 50 synthetic tested peptides as “binders” or “non-binders” according to NetMHCpan affinity prediction for binding to HLA-ABC. Of the 50 peptides tested by the ELISpot assay, 23 were considered to be binders. Four of these came from ETRAMP, 1 from Ubiquitin-Ribosomal, 2 from histones, 7 from 40S RP, one from the 50S RP, 3 from 60S RP, and 5 from uncharacterized proteins (Fig 2d). Twenty-seven peptides were considered non-binders, which included 2 peptides from ETRAMPs, 1 from Ubiquitin-Ribosomal, 4 from Histones, 6 from 40S RP, 12 from 60S RP, 1 from uncharacterized and 1 from the Don Juan protein (Fig 2e). When the ELISpot results of these two groups were compared, “non-binder” peptides had significantly higher positive responses (55.2%) than “binder” peptides that had only 32% reactivity (Fig 2f), indicating that most of the immunostimulatory peptides do not bind to the classical HLA-ABC.

Since non-HLA-ABC binder peptides had higher IFNγ responses and unusually long peptides were eluted from the HLA proteins of *Pv*-infected reticulocytes, we investigated whether this finding could be explained by a deficiency in the expression of antigen presentation pathway proteins in Retics that might favor the loading of unconventional peptides. Although the previously confirmed expression of transporter associated with antigen processing (TAP) protein^5^ suggests normal peptide transport into the endoplasmic reticulum, neither Tapasin nor Endoplasmic Reticulum Aminopeptidase 1 (ERAP1) were found to be expressed in either uninfected Retics (uRetics) or iRetics (Extended data fig. 5a)—suggesting an antigen processing abnormality that might affect peptide quality control and trimming in iRetics.

We next examined the expression of the non-classical HLA class I molecule E (HLA-E), which at steady state presents the HLA class-Ia leader peptide VL9 (VMAPRTLLL) to inhibit NK cell activation^23,24^. During infection, cell senescence and tumorigenesis, HLA-E complexes with non-canonical peptides derived from pathogens or neo-antigens have lower affinity to HLA-E^25,26^ and can be as long as 17-mers^27,28^. We found that HLA-E is expressed on Retics and upregulated on iRetics (Fig 2g), as previously reported for pan-HLA-I^5^. HLA-E/peptide complexes are also susceptible to immunoprecipitation during our immunopeptidome workflow, employing the pan-HLA-I (W6/32) mAb. In our immunopeptidome analysis, we did not find any human VL9 peptide, including sequence variants (SI. 2). Thus, we asked if our screened malaria peptides could be presented by HLA-E. PBMC from *Pv*-infected patients were stimulated with a pool of the 50 peptides or individual peptides under conditions in which we blocked pan-HLA-I with the W6/32 mAb or HLA-E with the VL9 peptide or their respective controls, isotype mAb or TH9 (TVCVIWCIH) peptide (Fig 2h–j). As expected, blocking with W6/32 mAb abolished the production of IFNγ by CD8^+^ T cells, while the isotype control did not. Importantly, HLA-E blocking by VL9 peptides reduced IFNγ production by CD8^+^ T cells by about 50%, while the negative control peptide TH9 had no effect (Fig 2h). In line with data from figure 1i, since CD4^+^ T cells did not produce IFNγ, the blocking conditions had no effect (Fig 2i). Similarly, single peptide stimulation under blocking conditions confirmed presentation by HLA-E, although some of these peptides were also concurrently presented by HLA-ABC (Fig 2j). Despite their ability to stimulate CD8^+^ T cells, the tested peptides had very low or no binding affinity to HLA-E*01:01 or 01:03, while some bound to HLA-A*02:01 (Extended data fig 5b). The widespread cross-reactivity of malaria epitopes recognized by *Pv*-infected donors likely is due in part to the pauciclonality of functional HLA-E alleles in human populations.

## Novel antigens are recognized by CD8^+^ T cells following *P. knowlesi* infection and liver-directed immunization

**Figure 3 |:**
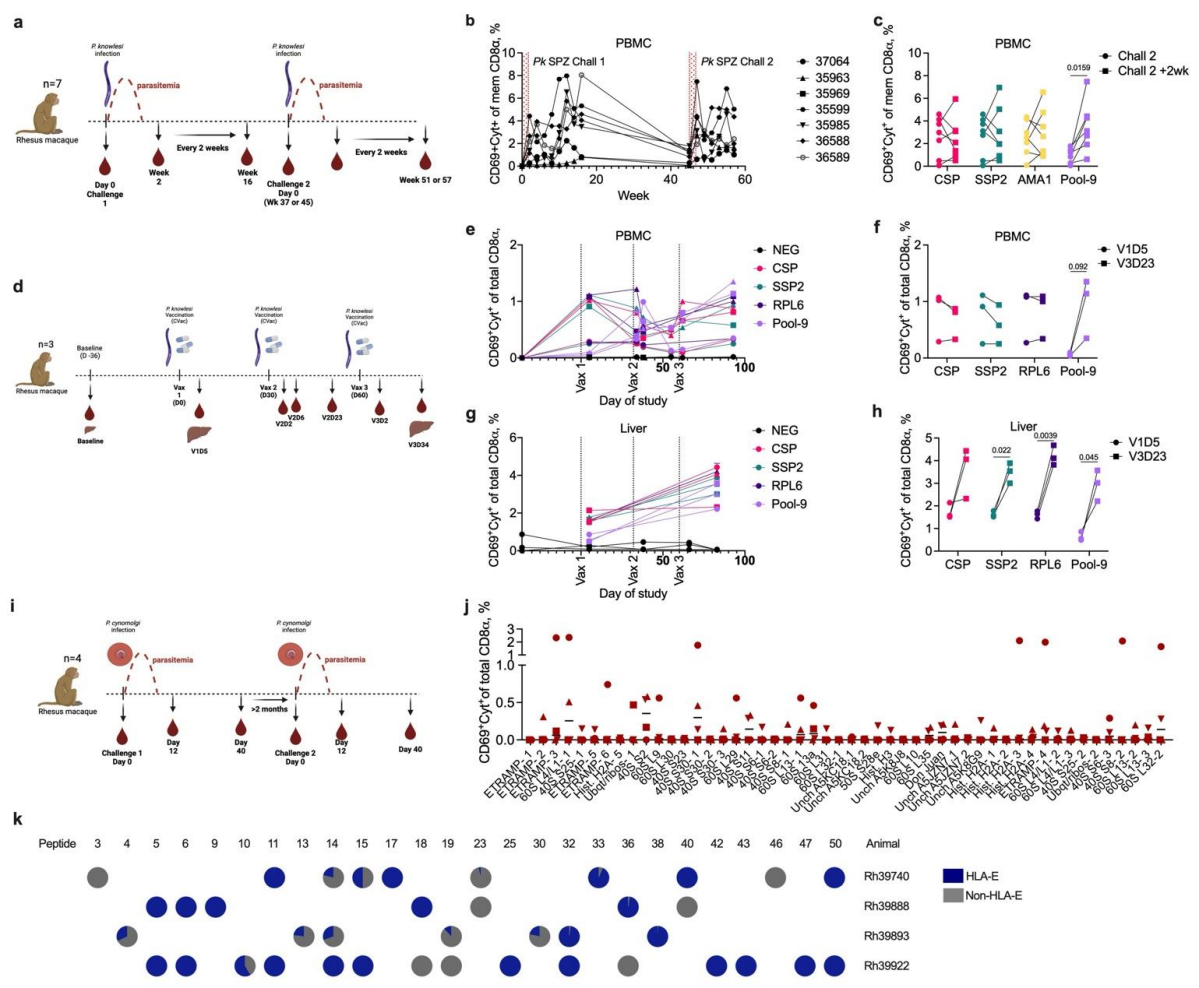
Novel *Plasmodium* antigens are recognized by T cells elicited during *P. knowlesi* (*Pk*) and *P. cynomolgi* (*Pcy*) infection of non-human primates. **a,** Schematic of repeated *Pk* sporozoite infection of rhesus macaques (n=7). Infection was initiated by intravenous injection of 2,500 purified, cryopreserved *Pk* sporozoites. After development of >2% parasitemia and treatment on days 10-12, blood samples were taken every two weeks to assess peripheral immune responses via ICS. This was repeated at either 37- or 45-weeks post-infection. **b,** CD8^+^ T cell responses to a pool of 9 *P. vivax* peptides conserved in *Pk* (Pool-9) in the peripheral blood of rhesus macaques following two sporozoite infections. The relative frequency of CD8^+^ T cell responses as defined by CD69 expression together with either IFNγ or TNFα is shown. Each animal is denoted by a unique symbol with timepoints connected by a line. Pink shading indicates the period of parasitemia. **c,** Pair-wise comparison of CD8^+^ T cells responding to overlapping peptides of indicated *Pk* antigens (CSP, SSP2, AMA1) or Pool-9 to assess boosting of CD8^+^ T cell response following a second infection. **d,** Schematic chemoprophylaxis vaccination (“Cvac”) where animals were injected with 50,000 purified cryopreserved *Pk* sporozoites under weekly chloroquine coverage plus coartem on days 7-9 post-infection. Immunizations were repeated at monthly intervals for a total of three immunizations. At indicated time points, peripheral blood with or without liver biopsies was taken to assess immune responses. **e,** Longitudinal CD8^+^ T cell responses in peripheral blood to overlapping peptides of indicated antigens or novel antigens. Each animal is denoted by a unique shape with timepoints connected by a line. **f,** Pair-wise comparisons of peripheral immune responses as in **c**. **g,** Same as in **e** employing liver lymphocytes. **h,** Same as in **c** employing liver lymphocytes. **i,** Schematic of repeated *Pcy* infection of rhesus macaques (n=4). Infection was initiated by intravenous injection of 36,000 cryopreserved *Pcy* Berok infected red blood cells. Blood samples were taken 12 days and 40 days after infection to assess peripheral immune responses. This was repeated >2 months post-infection. **j,** CD8^+^ T cell responses to each of the 50 conserved peptides in the peripheral blood of rhesus macaques 12 days after first in inoculation with *Pcy*-iRBC. Responding CD8^+^ T cells were defined as expressing CD69 and either IFNγ or TNFα, and frequencies of CD69^+^cytokine^+^ cells are shown after subtraction of responses from a DMSO control. Each animal is denoted by a unique symbol. **k,** the proportion of HLA-E-restricted CD8^+^ T cell responses was measured against a selection of conserved antigens after secondary infection with *Pcy*-iRBC. Antigens that induced a CD8^+^ T cell response after primary infection of each animal were selected for stimulation in the presence or absence of the HLA-E blocking peptide VL9. Total CD8^+^ T cell responses 12 days (39740, 39888, and 39992) or 40 days (39893) after secondary infection are shown with the proportion of HLA-E-restricted responses shown in blue and non-HLA-E-restricted responses shown in gray. Statistical analyses were performed by paired *t* test (**c, f, h**). Actual P values are shown.

Given the highly conserved sequences of our peptides across *Plasmodium* species, we next tested the immunogenicity of these peptides in a cohort of NHP (n=7) commonly used for malaria vaccination/challenge studies. NHP were infected with 2,500 cryopreserved *P. knowlesi* (*Pk*) sporozoites on two occasions and allowed to develop high parasitaemia each time (Fig. 3a). Given the limited availability of lymphocytes from these animals, we used a pool of 9 peptides (SI. 4: peptides 1, 4, 7, 20, 27, 31, 32, 33, 37, shown as “Pool-9” in figures) that were 100% conserved in *Pk* and highly immunogenic in humans, to track peripheral CD8^+^ T cell responses over one year during which two sporozoite challenges were conducted. All animals developed CD8^+^ T cell responses to this pool of peptides with varying degrees of magnitude and durability that persisted 16 weeks post-challenge and settled to a more consistent range at the time of second challenge 37 or 45 weeks later (Fig. 3b). These CD8^+^ T cell responses showed a pattern of anamnestic activation upon rechallenge with a boosting of CD8^+^ T cell responses to Pool-9 but not to overlapping peptides covering traditional antigens CSP, Sporozoite surface protein 2 (SSP2) and Apical Membrane Antigen 1 (AMA1) (Fig. 3c).

In light of published data supporting expression of these proteins during the liver stages, we next explored the immune responses to these antigens in the context of liver-directed immunization. Intravenous injection of live sporozoites under drug cover that quickly kills late liver or emerging blood stage parasites, called chemoprophylaxis vaccination (CVac), has been a potent means of generating a diverse and highly protective immune response to *Plasmodium* that can mediate high levels of sterilizing protection in mice, NHP and humans^8,29^. Here, we immunized NHP (n=3) with 5x10^4^ cryopreserved *Pk* sporozoites on three occasions one month apart and took serial blood samples and two liver biopsies to assess immune responses in the blood and liver (Fig. 3d). Similar to what is seen in humans^30^, all animals showed signs of functional liver-directed immunity as indicated by decreasing levels of parasitemia following each immunization (Extended data fig. 6a) and the development of anti-CSP IgG (Extended data fig. 6b). The CD8^+^ T cell response to three known pre-erythrocytic antigens (CSP, SSP2 and ribosomal protein L6 (RPL6)) and the “Pool-9” pool of nine novel antigens was monitored in the blood and liver. While responses to the three known antigens could be seen in the blood after the first immunization, these were not boosted by subsequent immunizations (Fig. 3e,f) similar to data in humans which fail to show a boosting of peripheral CD8 ^+^ T cell responses following live attenuated sporozoite ^29–32^. In the liver, CD8^+^ T cell responses were significantly boosted between vaccinations 1 and 3 to all antigens except CSP; this failure to demonstrate significant boosting against CSP is likely driven by a single animal which had unchanged responses to CSP from immunization one to three (Fig. 3g,h). Together, these results demonstrate that immunogenicity of at least a subset of these antigens are conserved in *Pk*-infected animals and are that CD8^+^ T cell responses are elicited and boosted during both blood and liver stage infection.

## Novel antigens are recognized by CD8^+^ T cells after repeated *P. cynomolgi* infection and are restricted to Mamu-E

The results with *Pv*, *Pf* and *Pk* led us to further investigate the CD8^+^ T cell response to these antigens in the non-human primate model of *Pv*—*Plasmodium cynomolgi* (*Pcy*). The NHP *Pcy* is a NHP malaria parasite that is phylogenetically closely related to *Pv* and, like *Pv*, forms relapsing dormant hypnozoites, demonstrates reticulocyte restriction *in vivo* and eventually can also infects humans^33,34^. A cohort of NHP (n=4) were infected twice with *Pcy* via direct blood stage inoculation, with at least 3 months between each infection (Fig. 3i). Patent parasitemia was confirmed for all the NHPs (Extended data Figure 6c). The CD8^+^ T cell responses to each of the 50 peptides was determined by intracellular cytokine staining (ICS) of peptide-stimulated PBMC during acute primary infection. Similar to our human data, at least 35 of the 50 peptides were recognized by CD8^+^ T cells from at least one animal, although magnitude varied greatly (Fig. 3j). During the acute phase of the second infection, PBMC from each animal were stimulated with those peptides that were recognized during primary infection with or without competition from the VL9 peptide. Importantly, the VL9 peptide also binds to the NHP homolog of HLA-E (Mamu-E)^35^. Like our previous data from humans (Fig. 2j), the CD8^+^ T cell responses to many individual peptides were fully or partially blocked in the presence of VL9—indicating a dependence on Mamu-E (Fig. 3k). Again, this was not strictly peptide-dependent as the CD8^+^ T cell responses to some peptides (14, 18, 36, and 40 – referenced in SI. 4) were VL9-blocked in one animal but not another. Together, these data indicate that a significant portion of the CD8^+^ T cells responding to conserved peptides after *Plasmodium* infection are Mamu-E-restricted.

## *Pv*-antigens induce specific CD8^+^ T cell responses in a murine model

**Figure 4 |:**
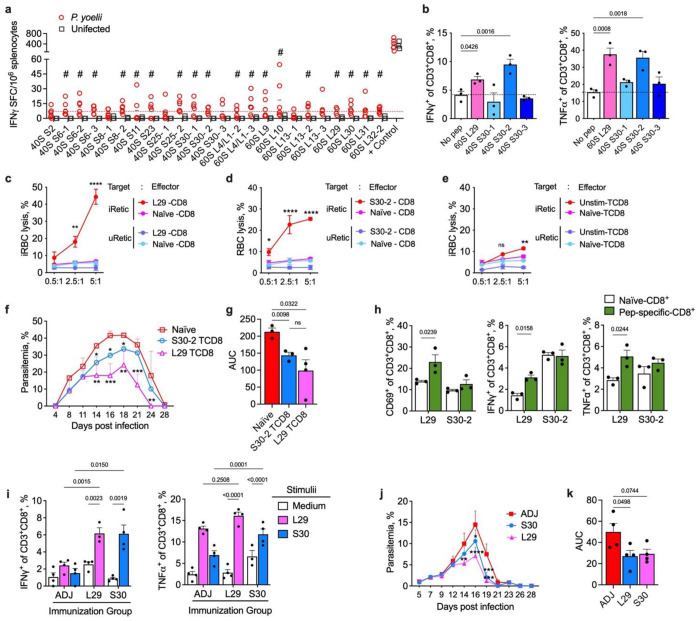
Antigen validation in the *P. yoelii* (*Py*) malaria experimental model. **a,** IFNγ ELISpot results for T cell responses to indicated peptides in *Py* infected mice. Mice were infected with 10^5^ *Py* iRBC. At 12 days post-infection (dpi), mouse splenocytes were isolated and incubated with selected peptides. Each circle represents one individual mouse. Red circles are *Py* infected mice (n=6), and black squares are uninfected mice (n=3). IFNγ production was measured by spot counting, and the results are expressed as SFC per 1x10^6^ splenocytes. Peptides were considered positive (#) when the group average was higher than 7 spots, shown by the red dashed line. **b-e,** Ag-CD8^+^ T cells were generated by *in vitro* expansion. Splenocytes were collected at 12 dpi and stimulated with each shown peptide in 12-day intervals. Cells were restimulated using Mitomicyn-treated APC from uninfected mice, pulsed with peptides. **b,** IFNγ and TNFα production by CD3^+^ CD8^+^ cells was evaluated by ICS, 12 days after a second peptide stimulation. **c-e,** *Py* iRetic and uRetic were labeled by CFSE and co-cultured with naïve CD8^+^ T cells, Ag-stimulated CD8^+^ T cells (**c**: L29.1 peptide; **d**: S30.2 peptide;) or unstimulated CD8^+^ T cells (**e**) at different effector: target ratios. Retics lysis was accessed by flow cytometry. **f-h,** Adoptive transfer of Ag-CD8^+^ T cells in the *Py* experimental model. **f-g** Mice (n=4) received 1.5x10^6^ Ag-CD8^+^ T cells at days 0 and 4, challenged with 10^5^ *Py*-iRBC, and followed for parasitemia for 28 days. **f,** Percentage of *Py* parasitemia after adoptive transfer. **g,** Area under the curve (AUC) relative to the parasitemia data. **h,** At 30 dpi, splenocytes were harvested and stained for activation marker (CD69) and cytokine production (IFNγ and TNFα) in CD3^+^CD8^+^ cells. **i-k,** Evaluation of immune protective responses induced by *P. vivax* ribosomal proteins (L29, S30). Mice (n=10) were immunized thrice with 10 μg of target protein plus adjuvant solution (18 μg CpG B344 adsorbed in Alum 30% v/v) at a 14-day interval and compared to the adjuvant alone group (ADJ). 21 days after the last immunization, blood samples were collected and spleens harvested (n=4) to assess the immune response to each target protein. On day 49, the remaining mice (n=6) were challenged with 10^5^ Py-iRBC, and the parasitemia was monitored for 28 days. **i,** ICS for IFNγ and TNFα production in CD3^+^CD8^+^ cells. **j,** Percentage of *Py* parasitemia in L29 and S30 immunized groups compared to adjuvant control. **k,** Area under the curve (AUC) relative to the parasitemia data. Statistical analyses were performed by two-way ANOVA test followed by Šídák’s multiple comparisons test. Actual P values are shown or represented by *P < 0.05, **P <0.01, ***P <0.001, **** P<0.0001.

The same peptides were also validated in a murine malaria model, using the reticulocyte-prone non-lethal *P. yoelii (Py*) 17XNL. Mice were infected with *Py*-infected reticulocytes (*Py*-iRetics), and splenocytes harvested at the peak of infection (12 days post-infection) for peptide stimulation. IFNγ-producing cells were analyzed by ELISpot (fig. 4a, Extended data fig. 7). About 75% of the peptides derived from RP stimulated IFNγ production at levels significantly above the uninfected control group (fig. 4a). For non-RP peptides, 1 out 6 ETRAMP, all 6 histones, and 5 out 6 other proteins peptides were immunogenic in the *Py* infection model (Extended data fig. 7).

To better understand the role of these responsive cells during the infection, we focused on two RP for the characterization of antigen-specific-CD8^+^ T cells (Ag-CD8^+^). The 60S RP L29 and 40S RP S30 were selected on the basis of the following features: (i) they were eluted from at least 5 immunopeptidome samples (SI. 2); (ii) they are expressed in blood and liver stage; (iii) they were identified as HLA-ABC binders by *in silico* analysis; (Fig 2a, Extended data fig. 1) (iv) they stimulated T cells from *Pv*- and *Pf*-infected patients, and *Pcy*-infected NHP, and *Py*-infected mice (Fig 1f, fig. 3j, fig. 4a).

We aimed to evaluate the potential role of Ag-CD8^+^ cells in eliminating *Py*-infected reticulocytes (*Py*-iRetics). To this end, CD8^+^ T cells specific to the peptides L29.1 and S30.2 were derived from the ribosomal proteins 60S RP L29 and 40S RP S30, respectively, by stimulating splenocytes from *Py*-infected mice with these peptides in the presence of IL-2 at 12-day intervals. After the second stimulation, CD8^+^ T cells specific for L29.1 and S30.2 peptides produced more IFNγ and TNFα compared to the unstimulated control (Fig. 4b). To validate if these Ag-CD8^+^ cells could specifically eliminate *Py*-iRetics, we performed *in vitro* killing assays employing varying effector:target (E:T) ratios^36^. Both L29.1-CD8^+^ and S30.2-CD8^+^ T cells were able to lyse *Py*-iRetics in a manner proportional to the E:T ratio; the specificity of this elimination was verified by also testing uninfected retics (uRetics) as target cells or naïve CD8^+^ T cells as effectors, which resulted in no appreciable killing activity (Fig. 4c–e). The CD8^+^ T cells from infected mice that were not stimulated with peptides but kept under IL-2 (unstim-CD8) displayed a minor, but significative killing only at the highest 5:1 ratio (Fig. 4e).

To test the role of these Ag-CD8^+^ cells *in vivo*, we adoptively transferred L29.1-CD8^+^, S30.2-CD8^+^ and naïve-CD8^+^ to naïve mice and then challenged with *Py*-iRetics. Both Ag-CD8^+^ cells were able to reduce the parasitemia burden, although L29.1-CD8^+^ controlled better (Fig. 4f,g). Upon infection, CD8^+^ T cells specific for L29.1 peptide were consistently activated and produced IFNγ and TNFα (Fig. 4h).

Lastly, we evaluated the ability of active immunization with whole recombinant proteins to elicit protective immune responses to *Py*. Wild-type mice were immunized three times at 14-day intervals with the recombinant proteins RP L29 or RP S30 in formulation with Alum and CpG adjuvants. Twenty-one days after the last immunization, the spleens of a subset of mice were harvested to assess Ag-CD8^+^ immune responses while another subset of mice were challenged with *Py*-iRetics (Fig. 4i–k). Immunization with either vaccine formulation induced Ag-CD8^+^ T cell activation as measured by IFNγ and TNFα (Fig. 4i). Both antigens reduced peak parasitemia and total parasite burden as measured by the area under the curve (AUC), while immunization with RP L29 was able to reduce parasitemia across multiple time points as compared to the adjuvant-only group (Fig. j,k). These findings highlight the potential use of the identified RPs as a malaria vaccine targets. Their expression across all parasite life cycle stages and high conservation among *Plasmodium* species suggest their ability to induce broad-spectrum protective immunity.

## Discussion

This study represents the first unbiased identification, characterization, and validation of *Plasmodium* HLA-I-bound peptides from infected reticulocytes, and the largest such data set for any stage or species of *Plasmodium*. We performed immunopeptidomics using high-resolution mass spectrometry and a bioinformatics workflow^11^ to determine the peptide repertoire presented by HLA-I molecules on the infected cell surface. Immunopeptidomics has been used previously to identify new vaccine targets for the development of novel immunotherapies and vaccines for cancer and infectious diseases^37–40^. A recent study on the discovery of new antigens against tuberculosis also using immunopeptidomics identified peptides presented by HLA-I and HLA-II, after which their immunogenicity was confirmed by ELISpot assay^41^. Three antigens were expressed in adenoviral vectors (ChAdOx1), alone or in combination, and assessed as vaccine candidates in a murine aerosol *Mycobacterium tuberculosis* challenge model. When administered together, the candidate vaccines provided significant protection in the lungs and spleen compared to BCG alone, validating this strategy for identification of novel vaccine antigen candidates^41^. Several studies have approached immunopeptidomics in parasitic infectious diseases context. CD4^+^ T cell antigens derived from *Leishmania* presented on mouse dendritic cell Major histocompatibility complex (MHC) II molecules were characterized by andem mass spectrometry (MS/MS), and the resulting antigens showed strong T cell responses in healed mice. Moreover, peptide was highly immunogenic in mice and humans^42^, demonstrating cross-species efficacy. Another study used *Trypanosoma cruzi*-infected murine fibroblasts to identify MHC class I-presented peptides employing an immunopeptidome workflow, which identified a small set of antigens that included mainly ribosomal proteins and heat shock proteins^43^.

The current study analyzed by immunopeptidomics *Pv*-infected reticulocytes from seven distinct donors. We identified 7,843 HLA-I-presented peptides of which 1,025 were derived from *Pv*. Of those, 453 were unique peptides mapping to 166 source proteins. Unexpectedly, the same peptide sequences or nested peptides were identified in different donors. Peptides that were common to 3-7 donors were selected for immunogenicity testing in a larger cohort of patients infected with *Pv*. Since many of the corresponding proteins are highly conserved in the different *Plasmodium*^44,45^ we also included samples from patients with a confirmed *Pf* mono-infection (i.e. not *Pf/Pv* mixed infection) in the ELISpot analysis. ELISpot results showed that T cells from patients infected with either *Pv* or *Pf* generated significantly higher responses to a pool of all 50 peptides or ribosomal peptides than T cells from NE-HD. Only *Pv*-infected samples induced higher ELISpot responses to ETRAMP and histones peptides; this result is likely due to the lower number of *Pf* cases in Brazil, our malaria study site, which limited our analysis to fewer samples compared to *Pv*. Some samples from E-HD, who had history of previous malaria, responded to peptide stimulation indicating some degree of lasting memory. Asymptomatic patients who control parasitemia without treatment also responded to the peptides. While this response was similar to that in symptomatic patients, investigation of specific antigens that may correlate with clinical protection in different geographical areas would clarify any potential protective role of Ag-CD8^+^ T cells across parasite species.

For several peptides tested in this study, we observed that T cells from different patients responded to the same peptide. Given the high polymorphism of HLA-ABC alleles in the Brazilian population^46^, one possible explanation for this observation is the ability of certain peptides to to various HLA-I molecules^47^. As expected, the HLA-ABC allele genotyping showed high diversity among the patients. In general, the HLA-ABC alleles found in these samples reflect the most frequent alleles in the Brazilian population^46^. Using this HLA-ABC typing data, it was possible to perform *in silico* peptide affinity prediction according to the alleles of each patient. From all *Pv* peptides identified in this study, 111 were HLA-ABC ligands of the alleles in the samples from which they were identified. Identical or nested sequences of HLA-ABC-binding peptides were found in different antigens. The 60S RP L29 had peptide ligands identified in a large number of the samples studied, with high affinity in five patients with different HLA-I alleles. Seven nested peptides of the 60S RP L13 were identified as ligands of HLA-I alleles from four different samples. The same was observed for peptides from other proteins with affinity for HLA molecules of three and two different patients. Even considering only peptides predicted to be HLA-ABC ligands, our analyses show that same *Pv* peptides can bind to different alleles.

The ELIspot results of NetMHC comparing MHC-I binder and non-binder peptides show that both had high IFNγ induction in *Pv* samples. Unexpectedly, non-binder peptides had a higher responder frequency in ELIspot compared to binder peptides. Since these peptides are longer than those usually considered as HLA-ABC ligands^48^, we investigated the antigen presentation machinery on reticulocytes. Our results show that reticulocytes are deficient in the expression of two of the major proteins responsible for peptide trimming and loading: ERAP1 and Tapasin^49^. A plausible hypothesis is that reticulocytes present long antigens that may be ligands of a non-classical HLA-I locus, namely HLA-E.

HLA-E belongs to the MHC complex and is considered one of the less polymorphic alleles of this family. In the Brazilian population, as is true for the global population, only two HLA-E alleles correspond to about 90% of the entire allele repertoire in this class, namely E*01:01 and E*01:03^50^. We found that CD8^+^ T cells to a number of peptides were restricted to HLA-E/Mamu-E by using VL9-blocking to disrupt HLA/Mamu-E presentation. While this strongly implicates HLA/MHC-E, further studies using newly developed blocking monoclonal antibodies, antigen presenting cells engineered with specific HLA alleles, and perhaps crystallography will be required to more fully understand the extent of HLA-E presentation as well as the observed promiscuity of some peptides.

The vast majority of peptides identified in this study were derived from essential housekeeping proteins such as RP and histone proteins. RP are essential for the protein synthesis machinery of prokaryotic and eukaryotic cells and are abundant in the *Plasmodium* blood and liver stages^51^. T cell responses to RP peptides were observed in both *Pv* and *Pf* samples. Interestingly, T cell responses to the same peptides were observed in mice infected with *Py* and NHP infected with *Pk* and *Pcy*. Together, this demonstrates that RP-derived peptides are targeted by the CD8^+^ T cell response across *Plasmodium* species. RP have been proposed as potential vaccine targets in several infectious diseases, including as a cross-species protective protein in different *Leishmania* species^52^, and as a CD4^+^ T cell-specific epitope in tuberculosis that can be boosted in mice previously primed with BCG mice^53^. Regarding the immunological potential of plasmodial RP, the *Plasmodium* RPL6 is the most potent, and perhaps the only *bona fide*, CD8^+^ T cell target identified to date. When targeted by tissue-resident CD8^+^ T cells, this antigen provided effective sterilizing immunity against *P. berghei* (*Pb*) sporozoite challenges in a mouse model^54^. Likewise, we demonstrated the functional role of CD8^+^ T cells specific to our newly-identified RP antigens using the *Py* 17XNL blood stage malaria model which, like *Pv*, infects reticulocytes. We showed that *Pv* antigen-specific CD8^+^ T cells can directly lyse *Py*-infected reticulocytes *in vitro*, and adoptive transfer of these cells can control blood-stage parasitemia. To further explore the broader spectrum of these antigens, we immunized mice with recombinant whole RP and observed a protective response against *Py* blood-stage parasitemia. Collectively, these findings highlight the potential of RP as targets for a malaria vaccine; future studies investigating the potential role of such a vaccine strategy in eliciting liver stage protection will be important.

Another protein class with potential as a vaccine antigen that was found in our immunopeptidomics screen was ETRAMPs (Early Transcribed Membrane Proteins). The two proteins from which the six ETRAMP peptides we investigated were derived are not highly conserved between *Pv* and *Pf*. Still, ELISpot assays show T cell responses in 48-82% and 28.5-71.4% of samples from patients infected with either *Pv* or *Pf*, respectively This finding suggests that there are conserved regions of the *Pv* ETRAMP that are able to induce a T cell response in *Pf* samples. In line with these findings, the ETRAMP also known as UIS3 (upregulated in infectious salivary gland sporozoites 3) has been studied as a vaccine candidate in the context of *Pf*. Humoral immunity and moderate levels of IFNγ were detected in mice immunized against *Pf*ETRAMP13/UIS3 using a ChAd63-MVA prime-boost, resulting in partial protection against challenge with transgenic *Pb* sporozoites carrying *Pf*UIS3 in a T cell-dependent^55^. Mice challenged with wild-type *Pb* (i.e. that had heterologous UIS3) were also partially protected, indicating the potential of ETRAMP T cells to mediated cross-species protection^55^. Addition of another partially protective antigen, *Pf*TRAP, further increased sterile protection^55^. Together with our studies demonstrating ETRAMPs as a potential vaccine target for vivax malaria, this suggests that ETRAMPs could be part of a multi-valent vaccine with the potential for cross-species protection.

Our study reveals that repeated *Pk* sporozoite infections can significantly boost CD8^+^ T cell responses to conserved peptides, particularly to a pool of 9 peptides highly conserved between *Plasmodium*, while responses to more traditional antigens like CSP, SSP2, and AMA1 remain relatively unchanged. Additionally, we observed that pre-erythrocytic immunization with CVac enhanced T cell responses not only to established protective antigens such as SSP2 and RPL6 but also to Pool-9 antigens, suggesting that CD8^+^ T cells targeting these newly identified antigens may play a crucial role in both blood and liver stage immunity.

In summary, we present here the identification, characterization and validation of *Pv* peptides associated to HLA-I of human reticulocytes. More than half of the identified antigens came from RP. These peptides were shown to be highly immunogenic in response to *Plasmodium* infection through *ex vivo* ELISpot assays, have high affinity for different HLA-ABC alleles, and strongly activate CD8^+^ T cells. Peptides found in *Pv* blood stage are derived from proteins also expressed in the liver stage parasite cycle. These antigens are highly conserved in *Plasmodium* species from *Pf* to *Pk, Pcy*, and *Py*, and are also expressed in the liver stage. As far we know, this is the first identification of *Plasmodium* antigens that are conserved across different species and different stages of the parasites down to the epitope level. By inducing broad-spectrum, cross-stage and cross-species immunity, these antigens could contribute to the development of vaccines effective against multiple *Plasmodium* species and life cycle stages, that can be called a universal malaria vaccine.

## METHODS

### Malaria infected patients and healthy donors

*P. vivax* (*Pv*)- and *P. falciparum* (*Pf*)-infected patients and endemic healthy donors were recruited in the Tropical Medicine Research Center (Porto Velho-Brazil). Asymptomatic *Pv*-infected donors were identified by active screening and followed up for clinical and parasitemia evaluation. Single species infections were confirmed by qPCR. Healthy donors from a non-endemic malaria region were recruited in Belo Horizonte, Brazil. All participants provided written informed consent for participation in the study, which has a protocol approved by the Institutional Review Boards of the Oswaldo Cruz Foundation and National Ethical Council (CAAE: 59902816.7.0000.5091).

### PBMC and *P. vivax* infected reticulocytes obtention

100mL of blood was collected from infected individuals and controls. First, mononuclear cells were separated from peripheral blood (PBMC). For this, the blood was diluted in a 1: 1 ratio, was gently added to a tube containing 15mL of Ficoll (GE Healthcare, USA) and the protocol previously described was followed^56^.

The red blood cell pellet resulting from PBMC purification was resuspended in RPMI culture medium in a 1:4 ratio. Diluted blood was added carefully into a 50mL tube containing Percoll 45% (GE Healthcare, USA) at 5x the volume of the red blood cell pellet. Samples were centrifuged for 15 minutes at 2000 rpm. After centrifugation, the reticulocyte interface was collected. Reticulocyte purified samples had 99% purity, identified by Giemsa-stained thin blood smear.

### HLA I- Peptide Purification

The detailed protocol to perform the immunopeptidomics using mass spectrometry for identification of MHC-bound peptides was previously described^11^. Briefly, *Pv*-infected reticulocytes were lysed in 500μl lysis buffer (1% Igepal, 300mM NaCl, 100mM Tris, pH 8.0) supplemented with cOmplete^™^ Inhibitor Cocktail (Roche). HLA complexes were immunoprecipitated using 1mg monoclonal antibody W6/32 against pan-HLA class-I complexes (GE healthcare) cross-linked to Protein A Sepharose beads using dimethylpimelimidate (DMP, Sigma). Cell lysates were incubated overnight with W6/32 beads. Beads were subsequently washed with 10 column volumes of 2 × 150mM NaCl in 50mM Tris, 1 × 450mM NaCl in 50mM Tris and 50mM Tris buffer without salt. Peptides bound to the HLA groove were released with 5mL 10% acetic acid. To separate peptides from the α-chain and β2-microglobulin, samples were further purified by HPLC (Ultimate 3000, Thermo Scientific) on a ProSwift RP-1S 4.6 × 50mm column (Thermo Scientific) by applying a linear gradient of 2–35% (v/v) acetonitrile in 0.1% (v/v) formic acid in water over 10 min. Samples were dried in speed vacuum and suspended in 20μl buffer A (1% acetonitrile, 0.1% TFA in water). Eluted peptide fractions were analyzed by nUPLC-MS/MS.

### nUPLC-MS/MS and data analysis

Ultimate 3000 RSLCnano System coupled with an Orbitrap Fusion Lumos Tribrid mass spectrometer (Thermo Scientific) were used to perform mass spectrometry. Sequence interpretation of MS/MS spectra were performed using a database containing all annotated human Uniprot entries (Uniprot- UP000005640 -09-2019) and *P. vivax* Uniprot entries (Uniprot- UP000008333- Version 08-2019) combined using PEAKS 7 (Bioinformatics Solutions Inc). Peaks score was used to indicate the statistical significance of the peptide-spectrum match. This value is converted to −10*log10, which means that a more significant match will have a higher −10lgP value. In this present study the Peaks score of 15 was used as a threshold. The length of 8-15 amino acid was restricted to select HLA-I peptides. Sequence homologies between *Pv* peptides and the human proteome were analysed on BLAST database. Peptides showing a minimum of two amino acids different from human were included. Same BLAST analyses were performed comparing peptides found in *Pv* samples to *Pf* proteome reference strain 3D7 (UP000001450- Version 08-2019).

### Transcriptome analysis of *Plasmodium spp.* public data

The transcriptome analyses were performed based on available open databases. Orthologous genes for the 166 *Pv* (Sal-1) proteins found in the immunopeptidomics analysis were established using PlasmoDB^57^. For *Pv* strain PVP01, there were 158 orthologous genes; 144 for the *Pf* strain Pf3D7; 126 for *Py* strain PY17X, 129 for *Pb* strain PbANKA; genes, 146 for PcyM, and 129 for PkNH.

The species and parasite stage gene expression was based on data deposited in PlasmoDB^57^ or in the following published articles. The liver stage gene expression of *Pf* and *Pv* were obtained from published data^18^. For all stages of *Pf*, there were two RNAseq data sets used^58,59^. Published data sets for *Py*^60^ and *Pk*^61^ were also used. For *Pb* the analysis was based on two published RNAseq Transcriptomes^62,63^. For Pcy two RNAseq Transcriptomes^64,65^ were used. For RNAseq analysis the cut-off used was less than 1 transcripts per millions (TPM) and for DNA microarray the cut-off were genes equal to zero.

### Synthetic peptides

Synthetic peptides were synthesised by GenScript, USA. Peptides were dissolved in DMSO to 5mM for ELISpot and spectral match validation.

### ELISpot assay

The *ex vivo* IFNγ ELISpot assays were performed using 5x10^5^ fresh PBMCs per well from *Pv*- and *Pf*-infected patients, endemic (E-HD), non-endemic healthy donors (NE-HD) and *Pv* asymptomatic donors. Cells were plated in duplicate into 96-well ELISpot plates (Merck Millipore) precoated with 4μg/ml anti-human-IFNγ (clone 1-D1K; Mabtech). A total of 46 peptides were tested at a concentration of 10μg/ml with stimulation for 18-20 hours at 37 °C under 5% CO_2_. Anti-CD3/anti-CD28 antibodies were used as positive control and cells cultured only with medium were the negative control (subtracted from all conditions). After cell removal, plates were developed for 2 hours in the same temperature condition as the stimulus in the presence of 0.2 μg/ml IFNγ, 7-B6-Biotin (Mabtech). Spot detection was performed following incubation for 30 min in the dark with BCIP/NBT Alkaline Phosphatase Substrate (Sigma). Spot-forming cells (SFC) were counted using the ImmunoSpot automated ELISpot counter. Samples were considered positive when SFC were equal to or greater than 30 SFC/million PBMC.

### PBMC intracellular staining

Total PBMC from *Pv* symptomatic patients, *Pv* asymptomatic individuals or healthy donors (HD) were stimulated with pools of peptides for the protein class subgroups (40S ribosomal, 60S ribosomal, ETRAMPs, Histones, other, or a pool of all the 50 peptides). No peptide was used as negative controls and anti-CD3/CD28 mAbs were employed as positive controls. After overnight incubation, brefeldin/monensin were added for 4h. The cells were harvested and stained with viability dye Zombie Yellow (Biolegend), then stained with anti-CD3, anti-CD4, anti-CD8 antibodies. Cells were fixed and permeabilized using Cytofix/Cytoperm (BD Biosciences) and stained with anti-IFNγ antibody. The samples were collected by FACSCelesta (BD Biosciences) and subsequent analysis performed using FlowJo software v10 (Tree Star). For HLA blocking assays, total PBMC from *Pv*-infected patients were blocked with anti-HLA-I antibody (W6/32) or VL9 peptide (for HLA-E blocking) for 30 minutes. Isotype antibody or TH9 peptide were used as blocking controls, respectively. Next, the pool with all 50 peptides was added to the PBMC culture for overnight stimulation and intracellular staining performed as described above. The negative controls consisted of a condition in which no peptide was added and anti-CD3/CD28 mAb were employed as positive controls.

### Spectral Match Validation

A spectral match validation experiment was performed to confirm that the peptide sequences were assigned correctly. Fifty synthetic peptides were selected, produced and compared to the original identified sequences, referred to as the biological peptides. Synthetic peptides were run in the same experimental conditions as biological peptides. The parameters used to confirm the sequence identity were the mass over charge [m/z] for each peptide, the charge number of each peptide [z], the intensity and distribution of each peak within each peptide sequence, and the peptide-specific retention time (RT). These were compared between synthetic and biological peptides and the sequences matched. (Extended Fig 2).

### HLA class I Genotyping

To determine the typing of HLA-ABC alleles from the samples used in the immunopeptidomics assay, the total genomic DNA was extracted from 5×10^6^ PBMC using the QIAamp DNA Mini Kit (QIAGEN) according to the manufacturer’s instructions. HLA four-digit typing was performed by Sanger sequencing on a 48-capillary ABI-3730 DNA analyser.

### Prediction of HLA-ABC binders

For data curation, biding affinity of all HLA-I eluted peptides were analysed using NetMHCpan4.0^22^ locally. Predictions were performed according to the HLA-ABC type of each sample. The best HLA-binding allele and the best score were identified for all *Pv* peptides. Peptides with a threshold 10% were considered as ligands.

### Immunoblot

Cell lysates of infected reticulocytes and uninfected reticulocytes, obtained by lysis in radioimmunoprecipitation assay buffer (Sigma-Aldrich) in the presence of complete protease inhibitor (Roche), were analysed by immunoblot probed for both ERAP1 and Tapasin (Abcam) after hemoglobin removal using HemogloBind (Biotech Support Group).

Each lane of reticulocytes was loaded with 50μg of protein, whereas the PBMC control sample contained 20μg of protein. The same membrane was probed for F-actin as a loading control. The secondary anti-mouse or anti-rabbit immunoglobulin-G antibody was detected by chemiluminescence.

### HLA-E staining

Total RBCs from healthy donors or *Pv* patients were stained with SYBR green (parasite nuclei), anti-CD235a-APC (RBC marker), anti-CD71-PE (reticulocyte marker) and anti-HLA-E-PE-Cy7, then analysed by flow cytometry. HLA-E expression was evaluated for the uninfected RBC population (CD235a^+^CD71^−^SYBR^−^), uninfected Retics (CD235a^+^CD71^+^SYBR^−^) or infected Retics (CD235a^+^CD71^+^SYBR^+^), in comparation to total RBC stained with isotype APC.

### *Pf* liver stage gene expression

In order to verify whether the proteins referring to the peptides found in *Pv* blood stage are also expressed in *Pf* liver stage, liver samples from humanized mice infected with the *Pf* strain NF135 were produced. . All procedures involving humanized liver mice were reviewed and approved by the Institutional Animal Care and Use Committee of the Oregon Health and Science University (IACUC protocol number: IP00002077). Mice were infected as previously describedl^66^. The highly infective strain PfNF135 was kindly produced and supplied by Robert Sauerwein^67^.

Livers from mice infected with 5 million sporozoites and were collected 5 days after infection . For the extraction of RNA, the RNeasy^®^ Plus Mini Kit (Qiagen) was used following the manufacturer’s protocol. For conversion of RNA into complementary DNA (cDNA) 1μg of RNA was used and the SuperScript^™^ IV First-Strand Synthesis System (Invitrogen) protocol was followed.

Five genes were quantified through the real-time quantitative polymerase chain reaction (RT-qPCR): 40S RP S25, 40S RP S30, 60S RP L30, 60S RP L13 and Histone H2A. In addition, two genes known to be expressed in *Pf* liver stage, CSP and TRAP, were used as positive controls. SYBR green was used as a fluorescent DNA intercalant, in addition, 5 μl cDNA and 300 nM of each primer were also added to the reaction (Extended data table 7). Tests were performed on the StepOne ^™^ Real-Time PCR System (Applied Biosystems) thermal cycler under the following cycling conditions: 1 cycle of 50 °C for 2 minutes, 1 cycle of 95 °C for 2 minutes, followed by 40 cycles of 95 °C for 15 seconds and 60 °C for 60 seconds.

Samples were normalized by calculating ΔCt (Ct of the target gene - Ct of the endogenous gene). *Pf* 18S rRNA was used as a normalizer. For the relative quantification of gene expression, the 2 -ΔCt calculation was performed.

### Non-human primate studies

Indian origin rhesus macaques (RM) were obtained from and housed within the Oregon National Primate Research Center (ONPRC) for these studies. Animals were pair-housed with a suitable partner whenever possible and had visual, auditory and olfactory contact with other animals. Rhesus macaques received commercially prepared primate chow twice daily and received supplemental fresh fruit or vegetables daily. Fresh, potable water was provided via automatic water systems. Physical exams including body weight and complete blood counts were performed at scheduled protocol time points. Animals were sedated with ketamine HCl with the addition of Dexmeditomidine and Atipamezole as a reversal agent for some procedures, including some blood draws and intravenous parasite challenge. For parasitemia assessment, capillary blood samples were obtained cage side by standardized ear prick procedures orcollected into EDTA-coated plastic vacutainer tubes. All experimental protocols and procedures were approved by the Institutional Animal Care and Use Committee at the Oregon Health and Science University under protocols IP00003877, IP00001114, and IP00001169. ONPRC is a Category I facility and the Laboratory Animal Care and Use Program at the ONPRC is fully accredited by the American Association for Accreditation of Laboratory Animal Care (AAALAC) and has an approved Assurance (#A3304-01) for the care and use of animals on file with the NIH Office for Laboratory Animal Welfare. The IACUC adheres to national guidelines established in the Animal Welfare Act (7 U.S.C. Sections 2131–2159) and the Guide for the Care and Use of Laboratory Animals (8th Edition).

### Infection and immunization of non-human primates with *Plasmodium* parasites

In studies of *Plasmodium knowlesi* infection, control animals from unrelated studies of rhesus cytomegalovirus (CMV) vaccines were utilized. These animals were either completely naïve (n=3) or immunized with non-malaria vaccine vectors (n=4). Each infection was initiated by intravenous injection of 2,500 purified, aseptic, cryopreserved sporozoites obtained from Sanaria, Inc. Cryopreserved sporozoites were thawed in a 37°C water bath for 30 seconds and resuspended in sterile PBS containing 1% human serum albumin to 1mL/animal. Sporozoites were injected within 30 minutes of thaw. Parasitemia was monitored daily by Giemsa-stained thin smear from day 6 onward until reaching 2% when treatment with artesunate (8mg/kg IM, once) plus chloroquine (15mg/kg once daily for three days). Parasitemia was monitored daily until cleared and twice weekly for four weeks to ensure treatment efficacy.

For studies of *Pk* immunization by chemoprophylaxis vaccination (CVac), animals were injected intravenously with 50,000 sporozoites/animal/dose with purified, aseptic, cryopreserved sporozoites obtained from Sanaria, Inc. Cryopreserved sporozoites were thawed in a 37°C water bath for 30 seconds and resuspended in sterile PBS containing 1% human serum albumin to 1mL/animal. Sporozoites were injected within 30 minutes of thaw. During the immunization period, animals were administered a 10mg/kg loading dose of chloroquine followed by 5mg/kg weekly beginning the week of immunization. In addition, animals were administered one tablet of Coartem twice a day (morning and evening) on days 6, 7 and 8 after each immunizing inoculation. Parasitemia was monitored via Giemsa-stained thin smear on days 7, 9 and 12 after each immunization with weekly monitoring thereafter. Blood was also collected into EDTA and nucliSENS Lysis Buffer (Biomerieux Inc. Cat# 200292) for qRT-PCR assessment of parasitemia.

For studies of *Plasmodium cynomolgi*, animals were infected with cryopreserved infected red blood cells of the *P. cynomolgi* Berok strain via intravenous injection. Glycerolyte-preserved stocks were thawed by in a 37°C water bath and 0.2x volume of sterile 12% saline solution added dropwise with mixing. After a 5-minute incubation at room temperature, 12 volumes of 1.6% saline were added dropwise while mixing. Cells were then pelleted at 800xg for 4 minutes, supernatant removed, and pellet washed twice with 10mL of 0.9% saline added dropwise while mixing. After final wash, the supernatant was removed, and cells resuspended in 1mL of RPMI/animal for injection. Parasitemia was monitored in peripheral blood by Giemsa-stained thin smears following ear prick or sedated blood draw into EDTA.

### Laparoscopic biopsy procedure

Animals were fasted overnight and sedated with an IM injection of 10 mg/kg ketamine HCl (Ketathesia^™^, Henry Schein Animal Health) with 0.015 mg/kg dexmedetomidine hydrochloride (Dexmedesed^™^, Dechra, Overland Park, KS). Following sedation, animals were intubated and placed on isoflurane (0.8–2%). All animals received 0.2mg/kg sustained release buprenorphine (Buprenorphine SR, ZooPharm, Fort Collins, CO) SQ for postoperative analgesia.

Once prepared for surgery, three incisions were made to allow for insertion of a 5mm camera port just caudal to the umbilicus and (2) 5 mm midabdominal instrument ports (right and left). A 5 mm Caiman vessel sealer (Aesculap, Center Valley) was used to perform a partial lobectomy (5-15gm) of the left medial or left lateral liver lobe. Incisions were closed with 4-0 monofilament absorbable suture and a local block with bupivacaine was performed. Following the procedure, all animals were reversed with atipamezole hydrochloride (Antisedan^®^, Zoetis, Kalamazoo), extubated, and recovered. For liver pinch biopsies the procedure was performed as described^68^.

### Intracellular cytokine staining

For T cell analysis, peripheral blood was collected in ACD anti-coagulant and PBMC isolated using a percoll gradient. For liver lymphocyte isolation, biopsies (4-20g) were stored in cold Servator B (Global Transplant Solutions) prior to isolation. Care was taken to process the tissue as quickly as possible after surgical removal (within 2 hours). Tissue was weighed and rinsed in base buffer (Hank’s Balanced Salt Solution (HBSS), 1.5% fetal bovine serum (FBS), 2mM CaCl2, and 5mM HEPES) followed by vigorous mechanical dissociation into a minced slurry using a vegetable chopper. Dissociated tissue was transferred to a 125mL vented Erlenmeyer flask containing 25ug/mL Liberase TL (Roche) in base buffer (75mL of Liberase buffer per 15g of tissue). The flask was agitated for 30 minutes at 250 rpm at 37°C and the digested tissue strained through a 70-micron cell strainer (Falcon). The resulting liquid suspension was centrifuged at 50xg for 2 minutes to remove hepatocytes. The supernatant was pelleted at 350xg for 5 minutes and pellet resuspended in ACK buffer (KD Medical) to remove red blood cells. Cells were washed, pelleted by centrifugation and resuspended in 10mL 20% Opti-prep (Iodixanol, Sigma) overlayed with 10mL PBS containing 2% FBS. NPCs were isolated from the gradient interface after centrifugation at 1500xg for 25 minutes with no brake.

Antigen-specific CD8^+^ T cell responses were measured via intracellular cytokine staining^69–71^. Briefly, PBMC were incubated with peptides for 1 hour, followed by addition of Brefeldin A (Sigma-Aldrich) for an additional 8 hours. Incubation without peptides served as background control. Stimulated cells were fixed, permeabilized and stained using combinations of the following fluorochrome-conjugated monoclonal antibodies: SP34-2 (CD3; Pacific Blue, Alexa700), L200 (CD4; AmCyan, BV510), SK-1 (CD8; PerCP-Cy5.5), MAB11 (TNFα; FITC, PE), B27 (IFNγ; APC) and FN50 (CD69; PE-TexasRed). Data was collected on an LSR-II (BD Biosciences) and subsequent analysis performed using FlowJo software (Tree Star). Lymphocytes were gated for CD3^+^ followed by progressive gating on CD4^−^ and CD8^+^ T cells. Antigen-responding cells in the CD8^+^ T cell subset were identified by their co-expression of one or more cytokines with CD69.

### Immunogenicity validation in *P. yoelii* rodent malaria model

For the animal study, procedures were performed following the National Council for the Control of Animal Experimentation (CONCEA). The protocols were approved by the Oswaldo Cruz Foundation (FIOCRUZ) Animal Experimentation Council (CEUA protocol LW-2/21).

C57BL/6 mice female mice, 8–10 weeks old, were purchased and maintained at the FIOCRUZ-Minas animal facility. Mice were infected with *P. yoelii* 17XNL PYGFP, a GFP-expressing strain purchased from MR4/ATCC (Manassas, VA, USA). Cryopreserved parasites were thawed and passaged *in vivo* twice before being used in experimental infections. A non-lethal inoculum of 10^5^ infected RBCs was administered intraperitoneally (i.p.).

### Mouse IFNγ ELISpot assay

To validate peptides in a murine model, mice were infected with 1x10^5^ *Py*-iRBC, a non-lethal strain that preferentially infects reticulocytes^72^. At 12 days post-infection (dpi), mice splenocytes were collected for *ex vivo* IFNγ ELISpot assay. 1x10^6^ cells were plated in duplicates into 96-well ELISpot plates (Merck Millipore) precoated with 5μg/ml anti-mouse-IFNγ (clone 1-D1K; Mabtech). Fifty peptides were tested at a concentration of 10μg/ml and cells stimulated for 20 hours at 37°C under 5% CO_2_. PMA (1μg/mL) and Ionomycin (100μg/mL) were used as a positive control and medium alone as negative control. After cell removal, plates were developed for 2 hours in the same temperature condition of the stimulus in the presence of 0.2 μg/ml anti-IFNγ, 7-B6-Biotin (Mabtech). Spot-forming cells (SFC) were counted using the ImmunoSpot automated ELISpot counter. The results are expressed as spot-forming cells (SFC) by 1x10^6^ splenocytes. Peptides were considered positive when the mean of the group was equal to or greater than 7 SFC/million splenocytes. The cut-off was established using the response of uninfected mice to each peptide, with the value calculated based on the average response ≥ 0 plus three times the standard deviation.

### Generation of epitope-specific Cytotoxic T cells (CTLs)

Generation of epitope-specific cytotoxic T cells was adapted from previous studies^73,74^. Mice were infected with 10^5^ *Py*-iRBC, at the peak of parasitemia (30-40% iRBC). The spleen was harvested, and splenocytes were seeded at 1x10^7^ cells in a 24-wells culture plate in 1.5mL RPMI 1640 supplemented with 10% Fetal Bovine Serum (complete RPMI; Sigma) plus 1μg/mL of each peptide. Twenty-four hours later 100U/mL of recombinant IL-2 (Biolegend) in 0.5mL complete RPMI was added to the culture. The restimulation occured every 12 days by 1:1 split of the cultures and a supply of 1ml of fresh medium supplemented with 5μg/mL of peptide, and 1×10^6^ Mitomycin-treated cells from uninfected mice, as stimulator cells^75^. One day later 100U/mL of IL-2 was added in 0.5mL complete RPMI. In the intervals between the restimulations, cultures were fed with fresh media containing 50U/mL of IL-2, when required.

### Cytokine production in mouse cells by Intracellular staining (ICS)

Twelve hours after the second stimulation, 1×10^6^ cells were harvested and cultured with Brefeldin A (Sigma-Aldrich) for 6 hours. Incubation without peptides served as background control. Cultures were washed twice with PBS, stained with Zombie Yellow (Biolegend), cells permeabilized and fixed using the Fixation/Permeabilization Kit (BD) and stained using the following anti-mouse fluorochrome-conjugated monoclonal antibodies: 17A2 (CD3; APC), 53-6.7 (CD8; PerCP-Cy5.5), XMG1.2 (IFNγ; Pacific Blue) and MP6-XT22 (TNFα; PE/Cy7) from Biolegend. Data was collected on an LSR-II (BD Biosciences) and subsequent analysis performed using FlowJo software (Tree Star). Lymphocytes were gated for Zombie-negative followed by progressive gating on CD3^+^, CD8^+^ T cells. Antigen-responding cells in the CD8^+^ T cell subset were identified by their expression of IFNγ and TNFα. Statistical analysis was compared to the unstimulated (unstim) group, using a two-way ANOVA test.

### Antigen-specific CTL Killing assay

The killing assay was performed based on methodology previously described^36^. Briefly, 12 days after the second stimulation (as above in Generation of epitope-specific CTLs), Ag-specific CTL cells were purified using CD8a^+^ T Cell Isolation Kit II (# 130-095-236; Miltenyi). *Py*-infected RBCs were isolated through magnetic separation using LS columns (130-042-401; Milteny) and then labelled with CellTrace CFSE Cell Proliferation (CFSE; Invitrogen). The purified CTL and labeled-iRBCs were co-cultured for 4 hours at 37°C, 5% CO_2_ followed by cell-specific staining using the anti-mouse monoclonal antibodies: 53-6.7 (CD8a; PerCP/Cy5.5), TER-119 (Erythroid Cells; APC/Cy7) from Biolegend. Precision Count Beads (Biolegend) were added to gate standardization. Data was collected on LSRFortessa^™^ Cell Analyzer (BD Biosciences), and subsequent analysis was performed using FlowJo software (Tree Star).

### Adoptive transfer of pep-specific-CD8^+^ T cells in *P. yoelii* experimental model.

To perform the adoptive transfer, twelve days after the second stimulation (see Generation of epitope specific CTLs) Ag-specific CTL cells were purified using CD8a^+^ T Cell Isolation Kit II (Miltenyi). Mice received 1.5x10^6^ pep-specific CTLs at days 0, before challenge and 4 post-infection. Mice were challenged with 10^5^ *Py*-iRBC, and the parasitemia was followed for 28 days by flow cytometry analysis. To monitor parasitemia, 3μL of a tail-blood sample was collected, diluted in 20 μL PBS and Heparin (Cristalia), then diluted 1:10 in FACS Buffer. Samples were collected on an LSRFortessa^™^ Cell Analyzer (BD Biosciences) until 100,000 RBCs were acquired. Infected-RBCs (GFP^+^) were gated based on GFP-expression by *Py* parasites, then parasitemia analysed using FlowJo software (Tree Star).

At endpoint assessment, splenocytes were harvested and stained for cell activation and cytokine production. Briefly, 1x10^6^ cells were incubated with 10μg/mL of specific peptide for 12 hours,followed by Brefeldin A incubation for 6 hours. Cells were then stained as described above using Zombie Yellow (Biolegend), Fixation/Permeabilization Kit (BD), and the following anti-mouse monoclonal antibodies: 17A2 (CD3; Pacific Blue), 53-6.7 (CD8; Alexa Fluor 700), GK1.5 (CD4; FITC), H1.2F3 (CD69; PE), XMG1.2 (IFNγ; APC) and MP6-XT22 (TNFα; PE/Cy7) from Biolegend.

### *P. vivax* Ribosomal Proteins immunization protocol

Mice (n=10) were immunized three times by subcutaneous (s.c.) injections with a 100μl solution composed of 10μg of target protein plus adjuvant solution^76^ (18μg of CpG B344 (Alpha DNA) and 30% (v/v) of alum rehydragel L.V. solution (Reheis) at14 day intervals. The ADJ group only received an adjuvant solution. Fourteen days after the last immunization, blood samples and spleen were collected from a subset of animals (n=4) for immune response evaluation. Cells were stimulated with 10μg/mL of each recombinant protein, and the ICS protocol was performed as described in the previous section. Lastly, 21 days after the last boost, mice (n=6) were challenged with 10^4^ Py-iRBC, and the parasitemia was monitored for 28 days, as previously described.

No statistical methods were used to predetermine sample size. The experiments were not randomized, and the investigators were not blinded to allocation during experiments and outcome assessment.

## Supplementary Material

1

## Data Availability

The data that support the findings of this study are available from the corresponding authors upon request. Further information on research design is available in the Nature Research Reporting Summary linked to this article.
