# Supplementary material for: Identification of cross-stage, cross-species malaria CD8+ T cell antigens
Source: Res Sq. 2025 May 30:rs.3.rs-6682089. Preprint. [Version 1] doi: 10.21203/rs.3.rs-6682089/v1 (PMC12154130; doi:10.21203/rs.3.rs-6682089/v1)
Supplement: 1 [file NIHPPrs6682089v1-supplement-1.pdf]

SUPPLEMENTARY INFORMATION (SI)

Supplementary information 1 | Clinical information

Supplementary information 2 | Identified peptides

Supplementary information 3 | *Plasmodium vivax* orthologous gene expression in parasite life cycle.

Supplementary information 4 | Selected peptides used for in vitro stimulation

Supplementary information 5 | HLA binding
